# Advanced Opacified Fiber-Reinforced Silica-Based Aerogel Composites for Superinsulation of Exhaust Tubing Systems in Semi-Stationary Motors

**DOI:** 10.3390/ma13122677

**Published:** 2020-06-12

**Authors:** Markus Heyer, André Berkefeld, Pascal Voepel, Barbara Milow

**Affiliations:** Institute of Materials Research, German Aerospace Center, Linder Hoehe, 51147 Cologne, Germany; markus.heyer@dlr.de (M.H.); andre.berkefeld@googlemail.com (A.B.)

**Keywords:** silica aerogel, fiber reinforcement, opacifiers, super insulation, boehmite particles, high temperature, generic part, manufacturing

## Abstract

Within this study, monolithic three-dimensional silica aerogel (SA) composite parts with super insulating properties are presented. A generic part based on fiber-reinforced (FR) silica aerogel for thermal insulation of the exhaust tubing system—to keep the exhaust gases as hot as possible to improve the efficiency of the catalyst system—was produced via a sol-gel-based molding process in combination with a supercritical drying using scCO_2_. A thermal conductivity of 16 mW m^−1^ K^−1^ was measured via a heat flow meter technique. In this manuscript, we present a full cycle of the material compound design, starting with fundamental material evaluation including aerogel optimization, opacifier influence, and casting process. The obtained generic part in shape of a half-shell for pipe insulation is characterized under real conditions.

## 1. Introduction

Since the diesel crisis, the emission rate of exhaust gases has become a relevant subject of discussion, and it has led to measures of strict control. Especially, diesel engines are being heavily scrutinized for their impact on the environment. Although the credibility of diesel engines is declining due to the surge in official forbiddance of their usage in city centers, the efficiency of diesel engines is typically higher than the efficiency of gasoline engines. Therefore, diesel engines are still considered for stationary, semi-stationary, and mobile applications. The catalysis of exhaust gases has become the main issue in the diesel controversy. Typically, selective catalytic reduction (SCR) is applied for the reduction of NO_x_ emissions. This catalytic system is located downstream of the exhaust system. With better insulation of the exhaust line, higher temperatures are achieved at the catalyst. As a result, pollutants, such as NO, are more selectively reduced to N_2_ at higher temperatures of around 600 °C [1,2]. With more efficient insulation materials, less space is needed to provide similar or even improved insulation performance which leads to lighter structures and, thus, saves weight and fuel consumption in comparison to state-of-the-art insulating materials. In recent years, aerogels have emerged as areas of focus among several research groups [3]. Given their nanostructured porosity, aerogels are perfectly suited for thermal insulation applications. As nearly every sol-gel-derived material can be transformed into an aerogel, the variety of aerogels as well as possible applications is enormous. Silica-based aerogels are amongst the most well investigated aerogel systems. Being purely inorganic, high temperature applications are addressable for these types of aerogels [3,4,5,6,7,8,9]. There are a variety of publications concerning the fundamental approaches to synthesizing and characterizing silica aerogels [3,4,5,6,7,10,11].

Typically, thermal conductivities of approximately 12–20 mW K^−1^ m^−1^ can be achieved in state-of-the-art silica aerogel (SA) materials [5]. Classical SAs are known to be very stiff and brittle and, therefore, difficult to produce in monolithic shapes for engineering component prototypes [12,13,14] which normally require mechanically stable materials for handling, further processing, and application. The brittleness of classical SAs has often been reported in the literature [15,16] and also been investigated by theoretical means also taking reinforcements into account [17,18,19]. To solve this problem, numerous fiber reinforcements (FRs) can be incorporated into the SAs [20,21,22]. For the manufacturing of half-shell prototype parts (as is aimed to prove the applicability of aerogels in this study), it is very important to improve the mechanical strength in terms of tensile and compressive properties. Aerogels without reinforcements would be unlikely to survive the targeted harsh conditions, such as vibrations, in a monolithic form and, therefore, also be unlikely to preserve the integrity of the composite part.

Silica aerogels have a very high temperature resistance compared to organic aerogels, but subject to a continuous application of temperatures in the order of 400 °C, the material begins to shrink [23]. As a result, the density and thermal conductivity of SAs increase. Furthermore, the sintering process leads to crack formation resulting in the complete failure of monolithic aerogels. Therefore, it is difficult to use pure SAs as engineering components over a continuous temperature of more than 400 °C or even above.

One way to increase the temperature stability of SAs is to produce hybrid silica alumina aerogels [24,25]. But the preparation of larger monoliths, designed with increased Al content, is difficult. Further problems rise due to the starting chemicals which are relatively expensive or carcinoid for health such as propylene oxide. A more inexpensive and simpler variant for the production of high-temperature-stable SAs is adding ceramic particles to the silica sol leading to a reduction of shrinkage of the aerogel at temperatures between 600 and 800 °C [26,27].

**Figure 1:**
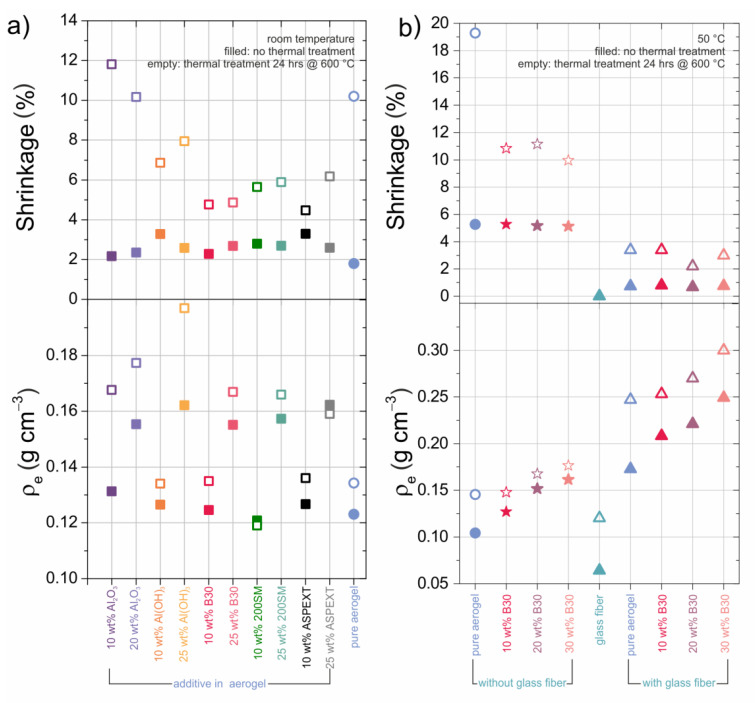
Density and radial shrinkage ratio of the pure silica aerogel (blue dots) and SAs combined with different ceramic opacifiers in varying concentrations before (filled) and after heat treatment for 24 h at 600 °C (unfilled). Two different synthesis temperatures were applied: room temperature (**a**) and 50 °C (**b**).

In this work, the embedding of different alumina species with different particle sizes in a SA matrix was examined. The addition of those particles increased the solid-state thermal conductivity in the entire aerogel. Accordingly, we aimed to work with the smallest possible amount of particle opacifier. Various ceramic particles, such as Al_2_O_3_, Al(OH)_3_, and boehmite, were incorporated into the SA and the influence of temperature resistance was examined (see Figure 1). Silica aerogels have a very low thermal conductivity, in the range of 14–16 mW K^−1^ m^−1^ because of the small pore sizes between 15 and 23 nm and high porosity above 96% [28]. As the thermal conductivity primarily consists of three contributions—the conductivity within the solid backbone (λ_s_); the thermal conductivity of the gaseous part (λ_g_); and the radiative (λ_r_) one—the addition of opacifiers (i.e., ceramic particles and mat of glass fiber) might increase the contribution of λ_s_, but on the other hand decreases λ_r_ at higher temperatures due to the ceramic’s behavior as an opacifier [29]. In order to take care of these effects, attention has to be paid to the geometrical structure of the opacifiers: The particles should be as small as possible so that the heat conductivity is only occasionally increased due to the ceramic particle.

Promising properties are obtained using tetramethyl orthosilicate (TMOS) and its corresponding solvent methanol [30] as a precursor, but its toxicity is challenging. Using an upscaling procedure to fabricate half-shell prototypes requires the substitution of these chemicals using less toxic and less harmful chemicals for the synthesis approaches [28]. Based on the chemical substitution, the material properties have to be optimized to guarantee the materials’ constant performance and properties. These changes may be useful to promote the upcoming industrialization of such aerogel-based composite components.

## 2. Materials and Methods

### 2.1. Materials

Tetraethyl orthosilicate (TEOS) was purchased from Merck (Darmstadt, Germany). Ethanol with a purity of 99% was purchased from Chemsolute (Th. Geyer, Lohmar, Germany) and used without further purification. Hydrochloride acid (2 M) was purchased from Bernd Kraft (Duisburg, Germany) and ammonium hydroxide solution (1 M) was purchased from Alfa Aesar (Thermo Fisher Schientific, Kandel, Germany). The different ceramic opacifiers used were: aluminum oxide (Al_2_O_3_) from Alfa Aesar (Thermo Fisher Schientific, Kandel, Germany); aluminum hydroxide from Sigma–Aldrich (Steinheim, Germany); boehmite B30, 200 SM, and ASPEXT all from Nabaltec (Schwandorf, Germany). The glass fiber mat “Insulfrax S Matte” was purchased from Insulcon GmbH (Neuss, Germany), and the Insulfrax S (Insulcon GmbH, Neuss, Germany) is a binderless needled blanket produced by mechanical needling of spun fibers.

The differences among the boehmite particles were mainly based on the particle sizes and the specific surface areas (see Table 1). Thus, the influence of these boehmite particles was examined more closely.

### 2.2. Methods

The shrinkage of the samples was calculated by measuring the diameter and height of the samples after supercritical drying and heat treatment at 600 °C for 24 h (in the following it is referred to only as “heat treatment”) and comparing with the original dimensions of the wet samples.

The porosity was calculated from the ratio of the bulk density and skeletal density which were determined by means of measurements with GeoPyc and AccuPyc (both from Micromeritics Instruments, Unterschleißheim, Germany).

For the aerogel–boehmite–glass fiber composites, a heat flow meter (HFM) device 436 Lambda (NETZSCH, Selb, Germany) was used for measuring the thermal conductivity of multi-material composites of larger length scales (190 mm × 190 mm). Additionally, the thermal conductivity was measured by the Hot Disk method with the thermal constants analyzer type TPS 2500 for smaller specimens of Hot Disk (Gothenburg, Sweden). Both measurements show an error of 2%.

The specific surface area (S_BET_) was calculated based on the Brunauer–Emmet–Teller (BET) method using TriStar II 3020 (Micromeritics Instruments, Unterschleißheim, Germany) nitrogen adsorption measurement. The samples were outgassed at 0.1 mbar and for 2 h at 120 °C.

The microstructure of the aerogels was investigated using a Merlin scanning electron microscope (SEM; Zeiss, Oberkochen, Germany). The non-conductive aerogels were sputtered for 90 s with platinum to obtain conductive samples for the SEM. The mechanical properties were determined using a universal testing machine 5566A (Instron, Darmstadt, Germany) with a load cell of 10 kN and a compression rate of 1 mm min^−1^. The measurements were carried out using cylindrical samples with a diameter between 38 and 52 mm (depending on the shrinkage).

The synthesis of the samples was based on an approach published earlier [6]. Here, the precursors TEOS, ethanol, water, and hydrochloric acid were mixed in a molar ration of 1:8:5.5:9.91 × 10^−5^ at room temperature for 3 h, then ammonia was added in a molar ration of 9.91 × 10^−3^. For optimization within this study, the synthesis temperature was increased to 50 °C, and the molar ratio of hydrochloric acid and ammonia was increased to 1.15 × 10^−4^ and 1.15 × 10^−2^, respectively, compared to the former approach, where the reaction temperature was set to room temperature. The opacifiers, if used, were added under vigorous stirring for 5 min after 40 min of hydrolysis of the silica precursors. After the addition of ammonia solution (45 min), the solutions were directly transferred into molds or infused into glass fiber mats. The last step had to be performed quickly, since the optimization drastically decreased gelation time. The samples have been prepared in cylindrical (disc-like) shapes. The glass fiber mat was cut into shape before infusion.

The samples were dried using a super critical CO_2_ extraction method at 115 bar and 60 °C after washing the samples in ethanol three times. The samples were characterized after drying as well as after a thermal treatment of 600 °C for 24 h. All samples (except for HFM and performance tests) were prepared in cylindrical (disc) shapes with a diameter of 50 mm (pre-shrinkage) and a height of approximately 19 mm. For HFM measurements, squared plates of approximately 190 mm × 190 mm were prepared, and for the performance test, a semi pipe was prepared.

## 3. Results

The most important motivation for the production of a SA glass FR composite is to minimize or even avoid shrinkage. It usually occurs during the supercritical drying process and/or during heat treatment for 24 h at 600 °C. The resulting huge shrinkage leads to cracked aerogels or detachment from the glass fibers [32]. Additionally, it decreases the aerogel’s porosity while increasing the density. Thus, the thermal superinsulation property of the aerogel was drastically changed (above 20 mW K^−1^ m^−1^ in preliminary tests) and aimed to be avoided in the presented study.

Preliminary tests were carried out leading to an observed overall, after drying and heat treatment, shrinkage of 5% to 12% in diameter and height. Therefore, we assumed an isotropic composite structure. For detailed values after drying and thermal treatment see Figure 1. The influences of different ceramic opacifiers in an amount of 0% to 30 wt.% in relation to TEOS are summarized in Figure 1.

In order to guarantee a homogeneous distribution of the particles inside the gel, a reduction in the gelation time was required to prevent sedimentation.

In the first step, this was achieved by increasing the temperature of the reaction from room temperature (RT) to 50 °C and slightly increasing the molar ratio of HCl from 9.91 × 10^−5^ (RT) [6] to 1.15 × 10^−4^ (50 °C) in relation to TEOS. With an increase in the temperature to 50 °C and the molar ratio of HCl to 1.15 × 10^−4^, the gelation time decreased from approximately 60 min to around 2 to 4 min thus leading to a negligible sedimentation of the opacifiers. First, preliminary tests were carried out with lower synthesis temperature (Figure 1a) already indicating the good shrinkage behavior of B30 added composites.

In Figure 1a, the shrinkage and densities of aerogel samples with various ceramic opacifiers, along with the influence of thermal treatment on the same samples by heating to 600 °C for 24 h, is shown. Figure 1b represents the influence of the new proposed synthesis route on the same parameters as in Figure 1a. The most promising silica–aerogel composite was realized using boehmite B30 as an opacifier and glass fiber mats for reinforcement.

The pure SA ((Figure 1a) blue-filled dots) was synthesized at room temperature (RT) and broke into several fragments after heat treatment. Therefore, this sample could not be analyzed. The pure SA synthesized at 50 °C (Figure 1b) had the highest shrinkage of approximately 20%, when measured in radial direction. Representative axial measurements did not hint to an anisotropic shrinkage.

No crack formation was observed for SAs with opacifiers such as Al_2_O_3_ and Al(OH)_3_ from 10 to 25 wt.%. If the amount of ceramic opacifiers was increased from 10 to 20 and 25 wt.%, the overall shrinkage of the sample decreased. The beneficial effect of Al_2_O_3_ already was described by Saliger et al. [27]. This effect was reproducible, with the addition of all considered boehmite particles. The shrinkage decreased for samples synthesized at RT by a factor of 2% to 5%, and the corresponding samples synthesized at 50 °C by 5% to 10%. This could be related to hydrogen bonding or even covalent bonding formed by condensation reaction among the hydroxyl groups of the SA surface and the boehmite particles. Several combinations were tested, and SAs with each embedded boehmite particles in combination with glass fiber mats showed the best performance. No dust formation, which is normally observed in aerogel–fiber composites upon tapping on the samples, was observed, and boehmite of the type “B30” exhibited the most homogeneous distribution in the aerogel. Amongst the three different boehmite powders, B30 was selected due to the fact of no observable sedimentation during gelation time. The silica sol and silica boehmite sol were infused into continuous glass fiber mats. Radial shrinkage of the resulting aerogel composites was reduced to a minimum of 1% to 4%. The density reached values between 0.25 g cm^−3^ and 0.30 g cm^−3^ after heat treatment.

**Figure 2:**
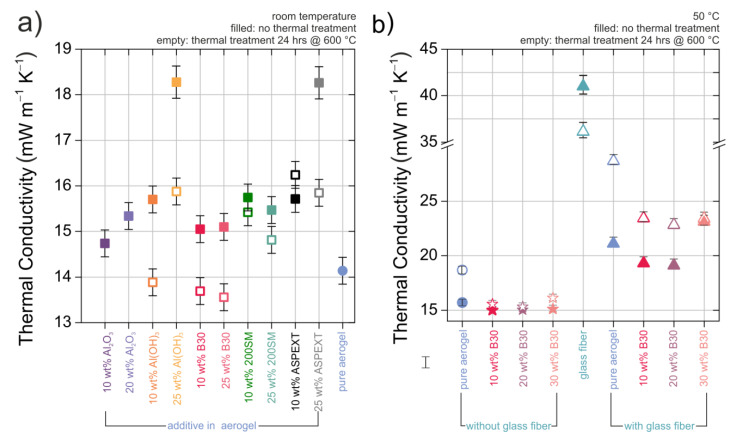
(**a**) Thermal conductivity of the pure silica aerogels (dots) (the thermal conductivity of the pure silica aerogels synthesized at room temperature (RT) could not be determined due to the fact of its decomposition into several fragments after heat treatment at 600 °C) and SAs combined with different ceramic (the silica–aerogel with the ceramic Al_2_O_3_ particles could not be measured after heat treatment, because the material broke into several pieces ) opacifiers and various amounts of them before (filled squares) and after heat treatment (unfilled squares) synthesized at room temperature. (**b**) Thermal conductivity of the pure silica aerogels (dot) and SAs combined with different ceramic opacifiers and various amounts of them before (filled triangles) and after heat treatment (unfilled triangles) synthesized at 50 °C (all measured via Hot Disk method).

The SA which was synthesized at 50 °C exhibited a slightly higher thermal conductivity of 18.5 mW K^−1^ m^−1^ after heat treatment compared to the aerogel before heat treatment (15.7 mW K^−1^ m^−1^) as depicted in Figure 2b. For the SA sample, which was produced at room temperature, the thermal conductivity could not be measured, because after sintering it disintegrated into several fragments (Figure 2a). Therefore, no data are shown; this is also the case for the Al_2_O_3_ additives. Based on increasing density induced by heat treatment and sintering, the thermal conductivity, λ_s_, of the material was increased. This effect was also observable while comparing the pure SA sample and the composite of SA with and without fiber reinforcement. The thermal conductivity increased from 21.1 mW K^−1^ m^−1^ to 28.7 mW K^−1^ m^−1^ (Figure 2b). The fiber reinforcement reduced the shrinkage (see Figure 1), but the thermal conductivity remained high after heat treatment. The as-received pristine mat of glass fibers without any opacifiers or aerogel had a relatively high thermal conductivity of 41.0 mW K^−1^ m^−1^, while after heat treatment, it reduced to 36.1 mW K^−1^ m^−1^ (Figure 2b).

The thermal conductivity of most composites with opacifiers based on the old approach was lower after the heat treatment (Figure 2a). However, the optimization of the syntheses led to a rather uninfluenced thermal conductivity by the addition of B30 if compared to the samples produced at room temperature (see Figure 2a,b). This might be attributed to a beneficial chemical bonding of the aerogel towards the B30 which might also explain the observed dispersive behavior. Also, the density was only slightly increased (Figure 1b) which possibly led to the suppression of the increase of solid-state thermal conductivity. The boehmite B30 particles (B30) were chosen in this test series as the best opacifier because of the composites’ very low thermal conductivity of approximately 15.0 mW K^−1^ m^−1^ before and 15.3 mW K^−1^ m^−1^ after heat treatment, very low shrinkage (Figure 1), and relative smoothness of the surface in reference to all other composites. The compatibility of B30 to the SA seems to be beneficial to prevent the thermal conductivity to rise after heat treatment. In addition, B30 was distributed more homogeneously in the SA than the other boehmites such as ASPEXT and 200 SM. The 200 SM particles formed a sedimentation layer at the bottom of the sample.

With the addition of B30, the shrinkage after heat treatment could be reduced to a minimum without an increase of thermal conductivity. Due to the higher density of the composite, the thermal conductivity after adding the opacifiers to the SA remained the same or was higher. The reason for increasing the thermal conductivity is that the connecting cross-sectional areas between two particles increase and, therefore, the heat transfer also increases by the larger areas [12,33].

**Figure 3:**
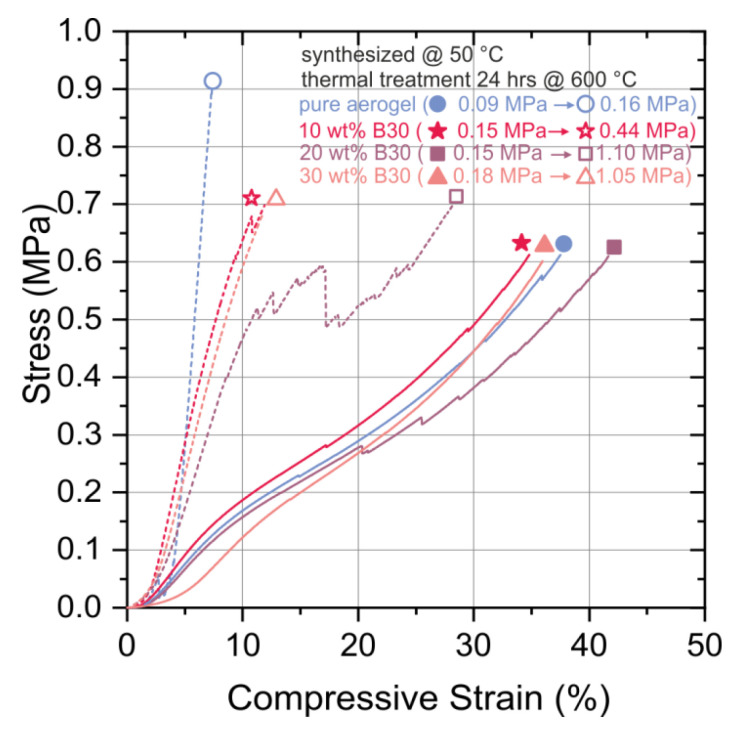
Compression curves of the pure SA (circle; blue line) and SAs combined with different concentrations of B30 before (full line and symbols) and after heat treatment (dashed line and empty symbols). All the specimens were synthesized at 50 °C.

Compression curves in Figure 3 exhibit an increasing Young’s modulus with an increasing amount of opacifier. The filled lines represent the pure SA and SA with B30 before heat treatment and the dashed lines after heat treatment. After heat treatment for 24 h at 600 °C, the Young’s modulus (Young’s modulus was calculated from the slope of the curve in the initial linear region for small strains) increased by a factor of 1.78 for the pure aerogel case, 2.91 for the 10 wt.% B30 case, 7.33 in case of 20 wt.% B30, and 5.83 for 30 wt.% B30 case, always compared to the pure SA. This increase in Young’s modulus is attributed to the condensation reactions or polycondensation (e.g., chemical reaction of hydroxyl groups with the release of water and formation of siloxane bonds) between the necks of two particles. The following types of particles are principally able to perform condensation reactions: SA to SA, B30 to B30, or SA to B30, and thereby the interface between two particles increases [34]. With the addition of B30, the uncondensed hydroxyl groups on the material’s surface of the SA condensate on the surface of the B30 and increased the Young´s modulus [33,35]. Furthermore, a part of the Young’s modulus increase was due to the shrinkage related to the increase in the density of the composite material after heat treatment [36]. The SA B30 composites without heat treatment can withstand higher deformation stresses than the sintered material because of its lower degree of cross-linking inside the material.

Summarized, it can be stated that the sintered SA B30 with 20 wt.% of B30 had a higher Young’s modulus by a factor 6.9 than the sintered pure SA. The main failure seems to be inside the aerogel matrix, as all specimens exhibited comparable strengths. However, the observed change after heat treatment indicated the mentioned influence of the opacifier.

**Figure 4:**
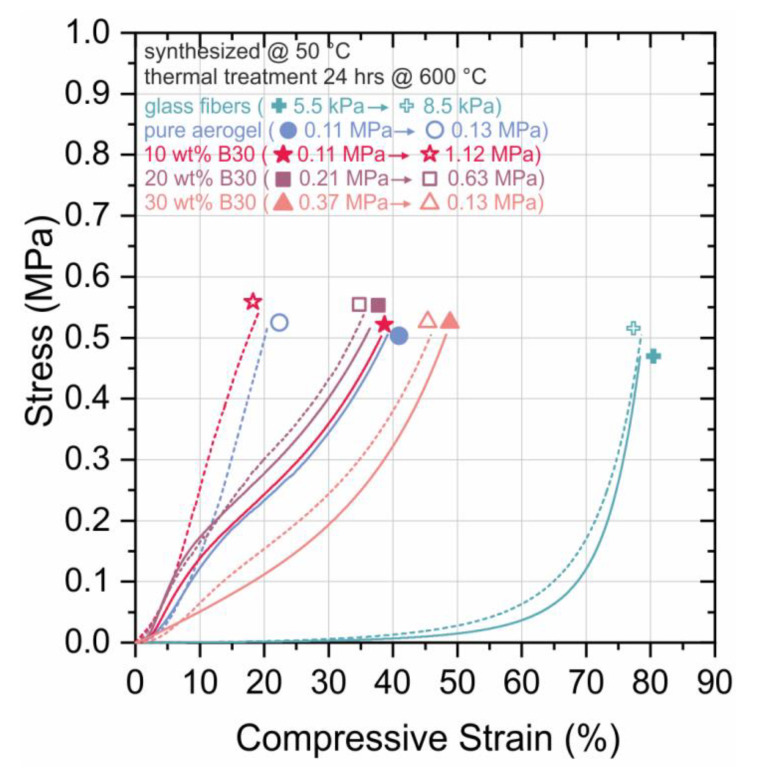
Compression curves of the pure mat of glass fiber (cross, green full lines) and SAs combined with B30 and glass fiber mat (other full symbols, full lines) and after heat treatment for 24 h at 600 °C (other empty symbols, dashed lines).

In Figure 4, the results of the compression tests before and after heat treatment of the fiber reinforced silica aerogel (FRSA) samples for 24 h at 600 °C are shown. The Young´s modulus increased after heat treatment for the SA without B30 by a factor of 1.18, for the SA with 10 wt.% B30 by factor of 10.2, and for the SA with 20 wt.% B30 by factor of 3.0; while for the FRSA with 30 wt.%, B30 reduced from 0.37 MPa to 0.13 MPa. The Young’s modulus of the pristine glass fiber mat increased after heat treatment by factor of 1.5.

The increase in Young’s modulus was due to the sintering behavior of the FRSA and B30. The addition of B30 leads to an increase in the Young’s modulus of the un-sintered samples. However, in the case where they were heat treated, the Young’s modulus decreased with an increasing amount of B30 for the FRSA samples.

It may be assumed that with an increasing amount of B30, the number of particles could be too high to be homogenously incorporated into the FRSA matrix. This gives hints that the multi-material system is weakened by bonding defects. The increasing amount of opacifier inhibits the condensation reaction between the aerogel and the glass fibers, as the contact between the glass fibers and the B30 become more prominent.

**Figure 5:**
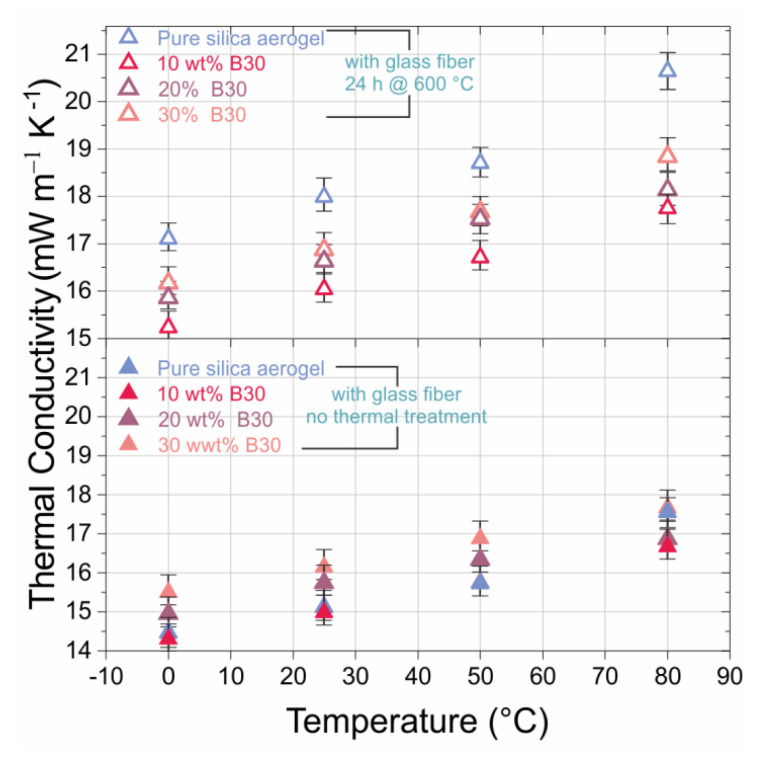
Temperature dependency of the thermal conductivity of fiber reinforced silica aerogel (FRSA) with B30 composites with and without heat treatment. All specimens measured via the heat flow meter (HFM) method.

The thermal conductivity of the FRSA–B30 composites increased with applied temperature (Figure 5). The amount of B30 not only changed the mechanical behavior of the samples but also the thermal conductivity after heat treatment. The addition of B30 led to its decrease. The performance of the untreated samples is shown at the lower half of Figure 5. In general, the FRSA outperformed most other composites in the low temperature range, exhibiting thermal conductivities of approximately 14.5 mW K^−1^ m^−1^. However, this relation was reversed at 80 °C and even more pronounced after heat treatment.

In that case, the pure FRSA exhibited the highest thermal conductivity over the whole temperature range which was attributed to the morphological and structural changes which have already been mentioned above. After heat treatment, the thermal conductivity of the samples rose with increasing amounts of B30. An addition of 10 wt.% B30 exhibited a thermal conductivity of 15.5 mW K^−1^ m^−1^ at 0 °C and approximately 18 mW K^−1^ m^−1^ at 80 °C.

In order to judge these results in comparison to the pure compounds, the thermal conductivities of the pure SA and the glass fiber mat are summarized in Table 2. As expected, the glass fiber mat exhibited a thermal conductivity of 30 to 35 mW K^−1^ m^−1^ which is higher by a factor of two compared to the pure SA. However, the composite based on the FRSA showed thermal conductivities in the range of approximately 15 to 20 mW K^−1^ m^−1^. Hence, the fairly well conducting fiber mat did not strongly influence the thermal conductivity of the composite. There, the conductivity was dominated by the aerogel despite having a mass ratio of the SA to the glass fiber mat of 0.4.

**Figure 6:**
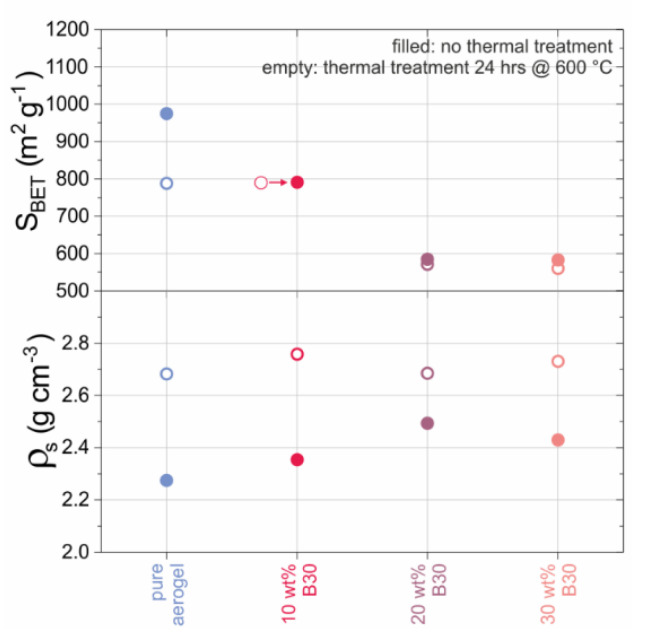
Comparison of the skeletal density and the specific surface area of the pure SA without and with various amounts of B30. (S_BET_ data point for 10 wt.% B30 after heat treatment was shifted to the left for reasons of visibility)**.**

In order to further investigate material changes during the high temperature application at 600 °C for 24 h, the relation between the amount of B30 and the samples’ densities as well as the specific surface areas are depicted in Figure 6. By adding B30 as an opacifier to the pure aerogel sample, the skeletal density increased from approximately 2.3 g cm^−3^ to more than 2.5 g cm^−3^. However, this effect was no longer present after heat treatment due to the reduced sintering effect of aerogel with embedded B30. The addition of B30 increased the skeletal density of the SA (Figure 2). This reduced the gaseous part and increased the solid-state dependent part on the thermal conductivity of the samples. The specific surface area was also affected by the addition of B30. The specific surface area decreased from almost 1000 m² g^−1^ for the un-sintered SA (800 m² g^−1^ for the sintered aerogel) to below 600 m² g^−1^ for the SA samples with the highest amount of B30. The B30 particles themselves had a specific surface area of 6 m² g^−1^ before heat treatment [31].

**Figure 7:**
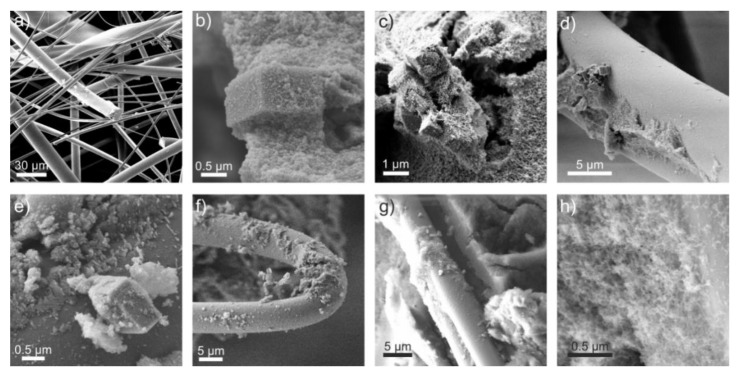
Representative SEM images of (**a**) a pristine glass fiber mat after heat treatment, (**b**) SA B30 composites after heat treatment, (**c**,**d**) FRSA–B30 composites before heat treatment and (**e**–**h**) after heat treatment at different magnifications.

Figure 7 depicts representative SEM images of (a) the glass fiber mats after the heat treatment for 24 h at 600 °C and (b) the aerogel B30 composites also after the heat treatment. The SA composite shows no significant optical differences compared to the non-heat-treated sample (c,d). From these micrographs, it can be inferred that the diameter of the glass fibers within the fiber mats were inhomogeneous. The diameter was determined to be within a range of 5 µm to approximately 20 µm. Moreover, the cuboid B30 particles were detected and the shape remained unaffected after sol-gel process. The surfaces of the B30 particles were partially covered with the SAs’ network–structure, and the nuclei seem to be formed on the surface (Figure 7b). The SEM images (Figure 7f–h) show a sintered SA B30 composite. It implies a rather good connection between the B30 and the SA. This could be due to the interactions of the surface hydroxyl groups of the B30 and the SA or due to the fact of a condensation reaction between both groups and formation of an atomic bonding. Nevertheless, the B30 particles seemed to form small aggregates in the SA, as it can be seen in Figure 7c. This effect might be caused by the agglomerating behavior of the particles during the reactions and a lack of dispersibility. Besides the attachment of the SA towards the B30 particles (Figure 7e), the growth of the SA on the fiber surface is also visible (Figure 7d,f). The SA surrounds the glass fibers and the structure of SA was preserved after heat treatment (Figure 7g,h).

These findings support the assumption that higher amounts of B30 decrease mechanical strength as proposed above.

**Figure 8:**
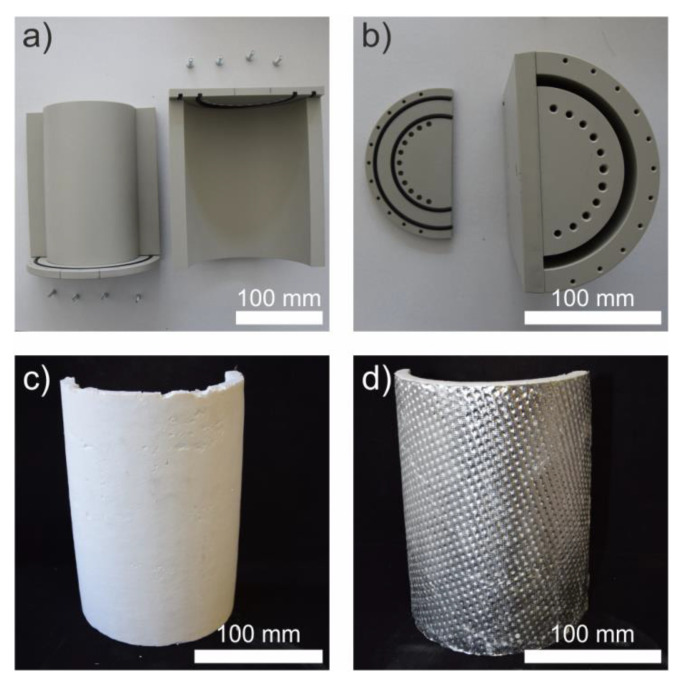
Photographs of the casting molds used to produce the (**a**,**b**) half-pipe-shaped composites and the finished half pipe prototypes after supercritical drying (**c**) without and (**d**) with an aluminum cover.

The best performing material with respect to thermal and mechanical properties as well as fabrication was FRSA with 20 wt.% B30 which was used to produce half pipes as depicted in Figure 8. These components were prepared by means of a casting process using a specially designed dismountable multi-part mold to receive the half pipe without damage (Figure 8a,b).

The final generic parts based on the developed process are shown in Figure 8c,d. In addition to the plain composite (Figure 8c), the half pipe made of the FRSA–B30 composites was in-situ covered with a perforated aluminum foil on the outside during the synthesis process (Figure 8d). The samples were 200 mm high and exhibited an inner diameter of approximately 125 mm with a wall thickness of approximately 10 mm.

**Figure 9:**
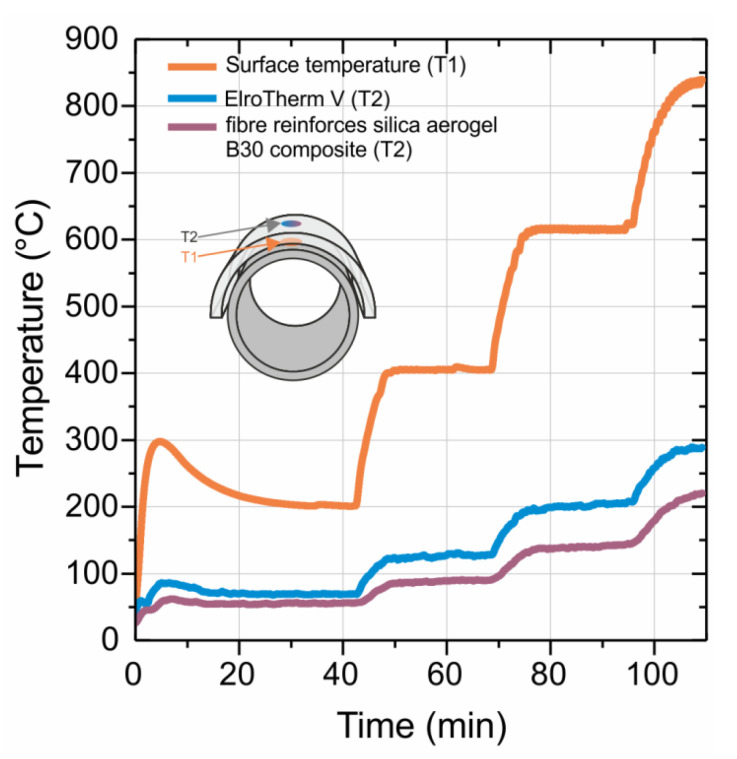
The surface temperature of the respective insulation material (T2) compared to the temperature of the heating device (T1).

The prepared samples were tested in comparison to a commercially available insulating material (ElroTherm V). The temperature on the surface of the insulation material was measured comparatively while being exposed to high temperatures induced on the surface of a hot tube. With increasing temperature, the difference in performance became more evident. After applying 200 °C on the surface of the hot tube, approximately 57 °C was measured for the commercial product, while 55 °C was measured for the FRSA–B30 composite after an equilibration time of 30 min. Additional tests were performed at higher temperatures. The corresponding plateau temperatures are summarized in Table 3. Thus, the temperature difference increased up to approximately 70 °C. In addition, the overheating of the tube within the first 20 min was better shielded by the aerogel composite (Figure 9).

## 4. Conclusions

We successfully prepared FRSA composites filled with various ceramic opacifiers. The gelation time of the sol was adjusted by the amount of hydrochloric acid and reaction temperature to prevent sedimentation of the opacifiers in the SA. The shrinkage of the SA was reduced after heat treatment for 24 h at 600 °C, from 20% to 5% by adding boehmite B30 particles. Additionally, the thermal conductivity of the SAs remained mostly stable.

In order to obtain a machinable and mechanically strong material, the SA with boehmite B30 particles was reinforced with a glass fiber mat. The Young’s modulus of these materials exhibited a strong dependency on the number of particles added, and the heat treatment applied to the material. An optimal amount was found in the region of 20 wt.% B30.

The reduction of the inner surface area through the sintering process was almost prevented by the addition of B30. Through the fiber reinforcement, a generic part with the shape of a half pipe was manufactured and tested on a test tube bench provided by ElringKlinger AG. At all measured temperatures, the outer surface temperatures of the SA composites were found to be lower by up to 70 °C compared to the commercially available ElroTherm V.

A new synthesis approach for a possible industrial production of silica aerogel half pipes was established. The new material has excellent heat insulating properties up to a temperature of 600 °C for continuous operation. For short-term use up to 24 h, the presented compound can resist temperatures up to 838 °C.

## Figures and Tables

**Table 1:** Particle sizes and the specific surface areas of the different boehmite-based ceramic particles [31].

Opacifier	D_90_ Particle Size	D_50_ Particle Size	D_10_ Particle Size	Specific Surface Area
	μm	μm	μm	m² g^−1^
B30	5	2.3	1	3
200 SM	0.6	0.3	0.2	17
ASPEXT	2	1.2	0.6	5

**Table 2:** Summary of the thermal conductivities of the pristine aerogel and the pristine glass fibers before and after heat treatment.

Temperature (°C)	Pristine Glass Fiber Mat (mW K^−1^ m^−1^)	Glass Fiber Mat after Heat Treatment (mW K^−1^ m^−1^)	Pristine SA (mW K^−1^ m^−1^)	SA after Heat Treatment (mW K^−1^ m^−1^)
0	29.3	29.6	13.43	23.51
25	31.9	32.2	14.14	26.89
50	35.6	34.1	16.32	29.53
80	38.6	36.6	20.41	40.68

**Table 3:** Different testing temperatures inside and outside of the testing materials on a technical demonstrator.

Temperature Heating Side (T1) (°C)	ElroTherm V (°C)	FRSA–B30 20 wt.% Composite (°C)
200	57	55
400	122	83
615	194	137
838	288	220
